# An Order-Sensitive Hierarchical Neural Model for Early Lung Cancer Detection Using Dutch Primary Care Notes and Structured Data

**DOI:** 10.3390/cancers17071151

**Published:** 2025-03-29

**Authors:** Iacopo Vagliano, Miguel Rios, Mohanad Abukmeil, Martijn C. Schut, Torec T. Luik, Kristel M. van Asselt, Henk C. P. M. van Weert, Ameen Abu-Hanna

**Affiliations:** 1 Department of Medical Informatics, Amsterdam University Medical Centers, Meibergdreef 9, 1105 AZ Amsterdam, The Netherlands; miguel.angel.rios.gaona@univie.ac.at (M.R.); m.abukmeil@amsterdamumc.nl (M.A.); m.c.schut@amsterdamumc.nl (M.C.S.); t.t.luik@amsterdamumc.nl (T.T.L.); a.abu-hanna@amsterdamumc.nl (A.A.-H.); 2 Amsterdam Public Health, Amsterdam University Medical Center, 1105 AZ Amsterdam, The Netherlands; k.m.vanasselt-2@umcutrecht.nl (K.M.v.A.); h.c.vanweert@amsterdamumc.nl (H.C.P.M.v.W.); 3 Centre for Translation Studies, University of Vienna, Gymnasiumstraße 50, 1010 Vienna, Austria; 4 Department of Laboratory Medicine, Amsterdam University Medical Center, De Boelelaan 1117, 1105 AZ Amsterdam, The Netherlands; 5 Department of Medical Biology, Amsterdam University Medical Center, Meibergdreef 9, 1105 AZ Amsterdam, The Netherlands; 6 Department of General Practice & Nursing Science, Julius Center for Health Sciences, Primary Care University Medical Centre Utrecht, 3584 CG Utrecht, The Netherlands; 7 Department of General Practice, Amsterdam University Medical Center, Meibergdreef 9, 1105 AZ Amsterdam, The Netherlands

**Keywords:** natural language processing, prediction models, machine learning, word embeddings, hierarchical attention network, primary care, lung cancer, early detection

## Abstract

**Background:** Improving prediction models to timely detect lung cancer is paramount. Our aim is to develop and validate prediction models for early detection of lung cancer in primary care, based on free-text consultation notes, that exploit the order and context among words and sentences. **Methods:** Data of all patients enlisted in 49 general practices between 2002 and 2021 were assessed, and we included those older than 30 years with at least one free-text note. We developed two models using a hierarchical architecture that relies on attention and bidirectional long short-term memory networks. One model used only text, while the other combined text with clinical variables. The models were trained on data excluding the five months leading up to the diagnosis, using target replication and a tuning set, and were tested on a separate dataset for discrimination, PPV, and calibration. **Results:** A total of 250,021 patients were enlisted, with 1507 having a lung cancer diagnosis. Included in the analysis were 183,012 patients, of which 712 had the diagnosis. From the two models, the combined model showed slightly better performance, achieving an AUROC on the test set of 0.91, an AUPRC of 0.05, and a PPV of 0.034 (0.024, 0.043), and showed good calibration. To early detect one cancer patient, 29 high-risk patients would require additional diagnostic testing. **Conclusions:** Our models showed excellent discrimination by leveraging the word and sentence structure. Including clinical variables in addition to text slightly improved performance. The number needed to treat holds promise for clinical practice. Investigating external validation and model suitability in clinical practice is warranted.

## Simple Summary

The research aims to improve early detection of lung cancer by developing better prediction models using free-text notes from doctor consultations, which capture the context and order of words and sentences. The authors created two models: one using only text and another combining text with clinical data. These models were tested on a large dataset of patients, with the combined model performing slightly better, accurately identifying high-risk patients who might need further testing. The findings show that these models could help doctors detect lung cancer earlier. The models should be validated in other populations before being adopted into clinical practice.

## 1. Introduction

Although diagnostic tools and new therapies have improved cancer survival, mortality remains 50% within 5 years after diagnosis. Lung cancer is among the cancers with the poorest prognosis, with a five-year mortality rate of 80%, according to Cancer Research UK (ancerresearchuk.org/about-cancer/lung-cancer/survival). In countries like the UK and the Netherlands, the General Practitioner (GP) acts as a gatekeeper, and in the Netherlands, more than 90% of lung cancer patients are diagnosed after a referral by their GP. However, referral often happens at an already advanced cancer stage, resulting in poorer outcomes. This highlights an urgent need for primary care to identify patients at higher risk of lung cancer at an earlier stage [1]. Currently, the median referral time is 13 days, though this varies widely (IQR: 1–484 days). Additionally, no systematic screening programs exist, meaning patients typically rely on symptom presentation to seek care. While alarm symptoms like hemoptysis prompt quicker referrals, other, less-specific symptoms often lead to delays in diagnosis and intervention.

Well-known prediction models, such as those by Hippisley-Cox et al. and of Hamilton et al. [2,3], are based on structured variables such as sex, age, smoking status, and/or (diagnostic) codes. Hippisley-Cox et al. reported an AUROC of 0.92 and of Hamilton et al. reported positive predictive values between 0.01 and 0.07. Aside from the fact that some variables, like family history or alcohol consumption, might not be consistently documented, the variables used pertain to known alarm symptoms and may appear only at an advanced stage of disease. These models might hence miss (non-trivial) clues manifested in earlier stages of disease. In particular, such models do not capitalize on free-text clinical notes that might possess such predictive information. Because of these limitations of current models, prediction models that use routinely collected free-text notes from primary care may improve early lung cancer detection [4,5].

This prospect has become viable in the last decade thanks to recent advances in Natural Language Processing. Unlike older approaches in which the syntax of the text is analyzed or word frequencies are calculated, new approaches map each word or text phrase to an “embedding”. An embedding is a vector in high-dimensional space (essentially a long sequence of numbers). The idea is that words that have similar or related meaning will also have similar embeddings. For example, the synonymous words ‘hepar’ and ‘liver’ will have similar embeddings, as well as the related words ‘coughing up blood’ and ‘hematemesis’. This approach is powerful as it captures the semantics (meaning) of words.

Various word embedding-based studies in medicine have been published, especially in intensive care for predicting in-hospital mortality [6]. Embedding-based approaches have also been reported for chronic disease prediction in general hospital admissions [7] and triage in emergency departments [8]. The vast majority of such studies focus on clinical notes in English. There is, however, a paucity of embedding-based studies on prediction models for cancer in primary care that use clinical notes, especially in non-English notes.

In a previous study, we looked at two embedding-based approaches to predict lung cancer in primary care at an earlier stage [4]. Surprisingly, we found that a simple approach—where all word embeddings of a patient were averaged, making it computationally not demanding—performed competitively with a method that accounted for word and sentence order [5]. Recent work has, however, shown that the order-aware approach that was applied can be significantly improved with a better optimization strategy [9,10].

Static word embeddings represent each word in a fixed manner, regardless of the surrounding context in which the word appears. Contextual word representations allow the same word to be represented differently depending on its surrounding words. In previous work, we also showed that approaches based on contextualized word representations for early lung cancer prediction using free-text patient medical notes from Dutch primary care physicians perform well when the number of non-cancer and lung cancer patients is similar. However, their performance deteriorates quickly when there are far fewer lung cancer patients than non-cancer patients [11] in the dataset, i.e., in cases of imbalanced data. Hierarchical attention networks [12] are simpler, static embedding approaches that are based on the same attention mechanism as contextual approaches [11] but perform better with imbalanced data [4].

The main aim of this paper is to develop and validate a state-of-the-art model for early prediction of lung cancer in primary care by capitalizing on (1) a hierarchical attention network that leverages the order and hierarchy among words and among sentences in clinical notes, and (2) a powerful optimization learning strategy based on target replication. In addition, we investigate the added predictive performance of using clinical variables on top of the clinical notes. If successful, such an AI-based model that leverages information in clinical variables and text may improve the early detection of lung cancer and positively impact the provision of lung cancer care.

## 2. Materials and Methods

We previously provided the deep learning for natural language processing (DLNLP) framework to standardize the reporting on NLP-based studies [6]. We use the DLNLP framework to report on the current study. The framework consists of two main components. The first is the approach of preprocessing and representing text, and the second is the approach for building a prediction model that turns the text representation into a probability of the event (lung cancer). The completed DLNLP form is available in the Supplementary Materials.

### 2.1. Patient and Data

In this retrospective observational cohort study, we used free text and structured routinely collected primary care data extracted from the Dutch primary care academic network of the Academic Medical Centers, University of Amsterdam. The dataset contains longitudinal extractions of electronic medical records of 250,021 subjects enlisted in 49 general practices between 2002 and 2021. The dataset contains de-identified patient data, including structured data like date of birth and laboratory results and coded data such as diagnoses and reasons for encounters. It also includes Dutch free-text consultation notes, which were de-identified by a custom version of the DEDUCE software (github.com/vmenger/deduce, last accessed on 26 March 2025). The GP, and occasionally a practice nurse, writes the free-text notes, usually during a consultation.

Based on date of birth, sex, and postal code, the primary care records of patients with lung cancer were linked to the Netherlands Cancer Registry (NCR, iknl.nl/nkr, last accessed on 26 March 2025) records, using a trusted third-party linkage procedure to comply with privacy regulations of Dutch and European law (https://gdpr.eu, last accessed on 26 March 2025). The NCR is a population-based cancer registry with detailed diagnostic and therapeutic data of over 98% of Dutch cancer patients. The diagnosis and month of diagnosis in the NCR were used as a reference standard.

### 2.2. Data and Predictor Extraction

First, we linked data by matching the GP electronic medical records (EMR) and National Cancer Registry (NCR). Then, we excluded patients without clinical notes before diagnosis and included only patients who joined at least 5 months before their diagnosis. Only patients older than 30 years were included. For patients with a lung cancer diagnosis, we used only entries of two years prior to 5 months before their diagnosis; for the other patients we used the same period of two years but up to one month before their last visit to the GP. We excluded patients who do not have clinical notes in that period.

Earlier research showed that in the Netherlands the median diagnostic interval for lung cancer was 49 days [1]. This refers to the time from the first presentation of symptoms which, in hindsight, are indicative of lung cancer, to the general practitioner, until the diagnosis is pathologically confirmed and registered by the NCR. Within this diagnostic interval, the median time from actual referral to registration of the diagnosis in the NCR is 21 days. The median time from first consultation with the specialist in the hospital to diagnosis is 8 days. Based on previous research [4,11], we only include data for training the models which was observed 5 months or earlier before diagnosis. This corresponds to blinding the model to, on average, 4 months before the actual referral to test its ability to predict lung cancer 4 months before the current referral.

The text is organized along five free-text fields in the SOEP format: S (subjective), describing the patient’s experience; O (objective), describing the doctor’s observation and the results of any research; E (evaluation), an explanation of the symptoms or diagnosis; P (plan), the action taken. An example of a generic patient note is given in Table 1. The first four fields are standard for Dutch EHRs. The prediction models take as input for each patient every visit as one sentence. All visits of each patient are aggregated (concatenated) while preserving the SOEP order. We excluded patients with very long notes that did not fit in the GPU memory. Specifically, we excluded patients whose total word count exceeded 99.7% (3σ) of the maximum allowable word count per patient. Words were represented as vector embeddings with the Word2Vec algorithm [13]. This algorithm learns representations such that words that are semantically related (e.g., ‘haemorrhage’ and ‘bleeding’) are represented by similar vectors of real numbers (embeddings). Word2Vec exploits frequent co-occurrence between words as a proxy of semantic relatedness. Our models used unigrams, which means that each vector embedding represented one word.

As structured predictors we used the age and sex of the patient. We also used the number of occurrences in the clinical notes of the following International Classification of Primary Care (ICPC) codes, known to be linked to lung cancer (who.int/standards/classifications/other-classifications/international-classification-of-primary-care): A04—Fatigue/weakness, B80—Iron deficiency anemia, B82—Other/unspecified anemia, P17—Nicotine dependence, R02—Dyspnea/respiratory distress attributed to airways, R05—Cough, R24—Haemoptoes, R95—Emphysema/COPD, T03—Reduced appetite, T08—Weight loss and L04—Chest symptoms/complaints [2,3,14,15].

### 2.3. Model Development

We developed two models for the prediction of lung cancer, which are based on hierarchical attention networks (HAN) [12]. For the first model (HAN-Text), only the GP free-text consultation notes were used. For the second model (HAN-Combined), we used both the GP free-text notes and the added (structured/coded) patient variables, together with the number of occurrences of each ICPC code related to lung cancer.

**Figure 1:**
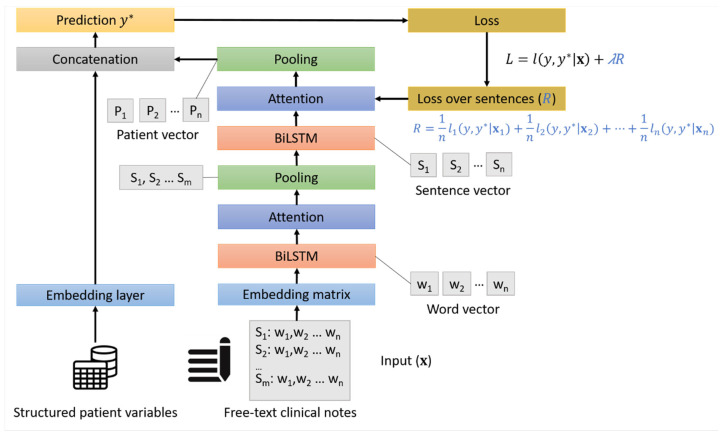
The architecture of the HAN models. Structured patient variables and concatenation are used only by the HAN-Combined model. In the HAN-Text, the last pooling layer is connected directly to the final prediction layer. The arrows represent the flow of the structured and free-text data in the model architecture.

The architecture of both models is shown in Figure 1. For handling text in both models, we adopted a two-layer architecture. The first set of layers represents words in a patient note (a “sentence” corresponding to one visit), and the second layer represents a sequence of such sentences for the final patient representation. These layers capture the order within and between sentences and are bidirectional recurrent neural networks, specifically bidirectional Long-Short Term Memory (BiLSTM) [16] because of their better handling of long-term dependencies. We also used an attention layer to capture dependencies between different parts of sentences [12] and we used target replication [9,10]. During training, target replication replicates the loss, which is the discrepancy between the prediction of the model’s current prediction and the observed value of the class (lung cancer or not), for each patient at intermediate steps (i.e., for intermediate visits). Target replication is represented mathematically in Figure 1 by the term *R* of the loss function, *L*, and it is controlled by a replication factor, *λ*, which is also a model parameter. In other words, an individual lung cancer probability is computed for every visit, and this intermediate discrepancy (i.e., loss) with the observed class is incorporated into the final loss. This has been shown to markedly improve the learning of the models.

For the HAN-Combined model, we used both the GP free-text notes and the structured patient variables, resulting in a total of 17 predictors. For these predictors, a hidden layer, with a rectified linear unit (ReLU) activation function, was used to capture possible interactions among these predictors. We combined the representation of free-text notes and the added patient variables via late fusion by concatenation. Late fusion aggregates the predictions from multiple models for different data sources and/or modalities, in this case one for the text and one for the structured data, into a final prediction [17]. Concatenation is the typical combination strategy of structured variables with text representation and means appending one vector representation to the other [6].

To understand the properties of text models, in addition to the two HAN-based models, we also developed four other competitive models that used text. Two models were variants of the HAN-based model but in one, the hierarchy of words and sentences was not used, and in the other, the attention mechanism was not used. The last two of the four models were based on the Phrase Skip-Gram (PSG) algorithm [4] or convolutional neural networks instead of the HAN. For these additional models, only the GP free-text consultation notes were used. These models are explained in Appendix A.

### 2.4. Model Evaluation

We randomly split the dataset in outcome-stratified training (60% for model development), tuning (20% for hyperparameter tuning), and testing (20% for unbiased model evaluation) sets. All models use the same training, tuning, and testing split.

We measured predictive performance in terms of: (1) discrimination, using the Area Under the Receiver–Operator Characteristic curve (AUROC), (2) the balance between the positive predictive value (PPV, also called precision) and sensitivity (also called recall), using the Area Under the Precision–Recall Curve (AUPRC); (3) the accuracy of the predicted probabilities, using the Brier score, which is the mean squared error of the predicted probabilities; and (4) calibration curves, which show how close the predicted probabilities are to the observed probabilities across the full probability range. Higher AUROC and AUPRC values mean better performance, whereas the lower the Brier score, the better the accuracy of the predicted probabilities. We also computed the sensitivity, specificity, positive predictive values (PPV), negative predictive value (NPV) based on a probability cut-off of 0.004 (essentially equal to the prevalence of lung cancer of 0.0039). The 95% confidence intervals of all measures were obtained on the predicted probabilities on the test set based on the percentile method with 1000 bootstrap samples [18]. We also calculate statistical significance based on the percentile bootstrap method on the differences in AUROC, AUPRC, and Brier score between the two models: we take the 2.5th and 97.5th percentiles of the differences and inspect whether 0 is included in the remaining 95% interval; if it is not, then there is a statistically significant difference between these results with *p* < 0.05.

Before training the HAN models, all words were first represented as embeddings based on the Word2Vec algorithms. These static embeddings are not fine-tuned on the class label. The Word2Vec embeddings were pre-trained only on the training set and not in the tuning nor the test sets.

## 3. Results

### 3.1. Patient Characteristics

After patient selection, 183,012 patients out of 250,021 were included, of which 712 (0.39%) were diagnosed with lung cancer. Table 2 describes the patient characteristics of our study population and statistics on the included consultation notes. Patients with lung cancer were older (34.4% between 60 and 70 years old and 29.6% between 70 and 80) than patients without lung cancer (17.4% between 60 and 70, and 10.1 between 70 and 80).

### 3.2. Predictive Performance of the HAN Models

**Figure 2:**
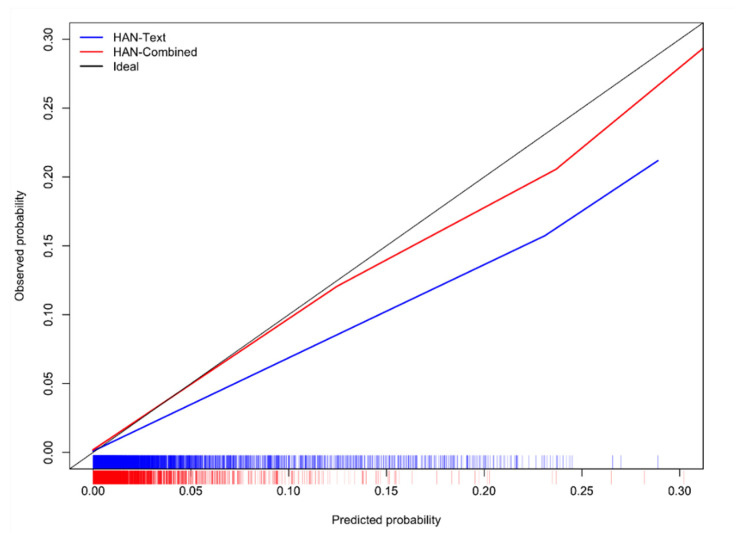
Calibration of the HAN-Text and HAN-Combined models (probabilities are trimmed at 30%).

Table 3 shows the AUROC, AUPRC, and Brier score of the models. The two models achieved similar performance (no statistical difference was detected). Both HAN-Text and HAN-Combined models achieved statistically significantly better results than the other 4 baseline text-based models for all these measures. These results are outlined in Appendix A. Table 4 shows the sensitivity, specificity, PPV, and NPV of both models. The HAN-Combined model achieved higher sensitivity and PPV than HAN-Text but lower specificity. The obtained NPVs are similar. Figure 2 shows the calibration curves of each model. They are obtained by using a locally weighted scatterplot smoothing (LOWESS) of the outcome on the predicted probabilities. The ideal line runs from (0, 0) to (1, 1). The HAN-Text tended to overpredict, while the HAN-Combined was well calibrated for most of the predictions.

### 3.3. Ablation Study and Comparison with Other Models

**Figure A1:**
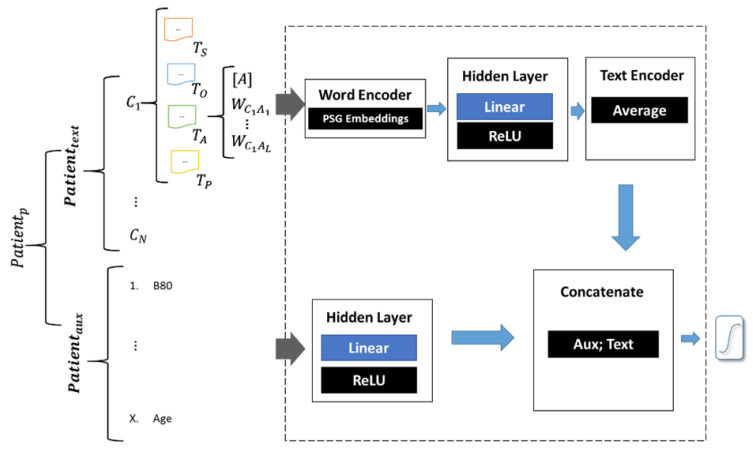
Architecture of the PGSNN model.

We compare the HAN-text model with additional models that can only use the GP free-text. Two models are variations of the HAN-Text model, and two other models are based on different neural architectures. A summary of the main model characteristics is given in Table 5, and the models are described in the following:

- **Hierarchical network (HN)**. It is a variation of the HAN-Text model where the attention layers have been removed.
- **LSTM**. A flat LSTM model, which does not exploit the hierarchy of words and sentences. Here, a BiLSTM layer represents a patient note (visit) and the final patient representation averages the representation of each note of the same patient.
- **Phrase Skip-Gram Neural Network (PSGNN)**. This model uses representation of 3-word phrases obtained with the Phrase Skip-Gram (PSG) algorithm [4]. These representations (embeddings) were then fed to a neural network prediction model through a hidden layer. The output of this layer was averaged to produce a single embedding that represents all the patient text. A logistic regression model was used to predict the probability of lung cancer. The architecture of the PGSNN model is shown in Figure A1 and described more in detail in Appendix A.
- **Convolutional Neural Network (CNN).** A two-layers CNN with max-pooling and target replication as performed by Grnarova et al. for mortality prediction [9]. The model is described more in detail in Appendix A.

The results are reported in Table 6 and Table 7. The AUC of the HAN model is statistically significantly higher than all other models with *p* < 0.05. The HAN AUPRC is statistically significantly better than all other models, apart from HN, with *p* < 0.05. The HAN Brier score is statistically significantly better than the LSTM and PSGNN models with *p* < 0.05.

## 4. Discussion

### 4.1. Main Findings

Our results demonstrate that capturing the order and hierarchy of words and sentences leads to excellent model AUROC at four months before the current referral date. Furthermore, including structured variables seem to improve the predictive performance, albeit not statistically significantly so. The optimization strategy based on target replication was crucial in learning a performant model. Such an AI-based model that leverages information in both clinical variables as well as text has the potential to improve the early detection of lung cancer. Specifically, the prevalence of lung cancer in our population (30+ years, general population enlisted in general practice) was 0.4%. The PPV of 0.034, although seemingly low, does hold promise for clinical practice, implying that to detect one patient with lung cancer 4 months before the current referral (5 months before diagnosis), 29 high-risk patients would need additional diagnostic testing. The model sensitivity shows that the model adequately identifies 93% of all patients with lung cancer around 4 months earlier than present practice. Given the stage-related prognosis of lung cancer, this can lead to a clinically relevant improvement [19].

Our AUPRC indicates room for improvement. AUPRC was until recently considered especially important in class imbalance scenarios like ours, where there are many fewer patients with lung cancer than non-cancer patients. However, the recent literature shows that AUPRC is not always the best metric under class imbalance [20]. McDermott et al. showed that while optimizing for AUROC equates to minimizing the model’s false positive rate (FPR) in an unbiased manner over positive sample scores, optimizing for AUPRC equates to minimizing the FPR specifically for regions where the model outputs higher scores relative to lower scores. They recommend using AUROC for context-independent model evaluation, for deployment scenarios with elevated false negative costs and for ethical resource distribution among diverse populations. They do advise AUPRC for reducing false positives in high-cost, single-group intervention prioritization. We report both measures.

Our results are better than the current practice. Furthermore, GPs do not need to refer every high-risk patient to an oncologist. They can first perform an intermediate step, such as a follow-up in 2 weeks and/or a chest X-ray, which is a cheap and non-invasive intervention. Thus, the cost of false positive is relatively low, and AUROC remains a relevant metric.

Regarding the comparison of HAN-text with other models, one notable result is that the HN model perform close to the HAN-text. This result could be explained by several factors. The hierarchy in the HN may already capture the necessary contextual dependencies for the task, and the benefit of attention could be small. The notes are organized in the clear SOEP structure and are rather short documents. Thus, words might have uniform importance (all parts of the input contribute equally to the output) and attention could have a small effect. Furthermore, for shorter sequences LSTMs can often memorize or encode all relevant information without needing attention to “focus” on specific parts. Finally, the higher-level LSTM (sentence-level) might already act as a “soft attention” mechanism by selectively propagating important information forward. For instance, the forget gate in LSTMs can discard irrelevant information, mimicking attention-like filtering or the final hidden states of the word-level LSTM may already summarize key features, reducing the need for explicit attention.

### 4.2. Related Work

Analyses of medical texts received increasing attention in recent years [6,21,22,23]. Most often conventional natural language processing (NLP) techniques like entity extraction and enrichment of text with term systems were used in primary care [24,25,26]. Beyond primary care, but still using routinely collected data, topic modeling and extraction of clinical concepts [27] and unsupervised text representations [28] were used. While these studies compare different prediction models, and some report on internal validation (using, e.g., cross validation), none has performed (or report on) statistical significance tests on the added performance value of structured variables in addition to text in the different models.

Not many NLP models for lung cancer prediction have been proposed. In previous work, we compared two embedding-based approaches to predict lung cancer in primary care [4,5]. A simple approach that averaged all word embeddings was competitive to one that was aware of the word and sentence order. In our current study we showed that a better optimization strategy, namely target replication, enables the more complex method to better exploit its potential and outperform simpler methods (see comparison in the Appendix A).

We also previously investigated different NLP approaches based on contextualized word representations for the problem of early prediction of lung cancer using free-text patient medical notes of Dutch primary care physicians while controlling data imbalance [11]. Contextual word representations enable the representation of the same word differently depending on its context in terms of its surrounding words. While contextual word representations perform well when the number of non-cancer and lung cancer patients are similar in the data (balanced data), their performance quickly degrade in realistic scenarios, when lung cancer patients comprise a tiny percentage of the sample (with a prevalence of 0.39% as in this study).

Our models in the current study are based on HAN, an advanced recurrent neural network which leverages the hierarchy of words and sentences and attention, but we also tested simpler recurrent neural networks, a convolutional neural network and a simple approach based on dense word embeddings and logistic regression similar to the one that was effective for colorectal cancer [29]. All these models performed worse than our HAN models (see comparison in the Appendix A).

More broadly, NLP has also been applied to other clinical domains than oncology. Many NLP prediction models focus on intensive care, also because of the availability of the publicly available data of the MIMIC and eICU datasets [6]. Much work used static word embeddings as we did, such as [9,30,31,32,33]. Static word embeddings represent a given word in the same way no matter which words surround it. Contextual embeddings are emerging [34,35,36]. Recurrent neural networks, also employed by us, are commonly adopted, e.g., [30,33,37]. Target replication was previously used with a convolutional neural network [9], while we employed it in a hierarchical recurrent neural network with attention.

### 4.3. Strengths and Limitations

This study has several strengths. We used real-world routinely registered consultation notes of general practitioners of a representative Dutch population of all patients older than 30 years with a general practice record including a history of at least two years with at least one consultation note in general practice. The database was large and included patient records from 49 general practices. In addition, the cancer diagnosis was validated by linkage to the Netherlands Cancer Registry. General practitioners mostly will code data only when they think the data are relevant, and therefore coded data will only form a selection of available information based on the current knowledge. By using free text, we avoided this coding bias, and much of the available information is captured, not just information which is considered relevant a priori by GPs, who generally tend to record diagnoses rather than other information, such as symptoms. We included temporal information of the consultations (order of consultations, word and sentence) as well as hierarchy of text (word and sentence). Our model achieved excellent results and is much cheaper to compute than relying on recent approaches, such as using large language models. Finally, this is one of the few studies that investigates the use of word embeddings for text in a language other than English (in our case, Dutch) in primary care.

Our approach also had limitations. One limitation is that international differences in data structure, patient presentation, meaning of words and GP’s registration may limit the usability and applicability of a developed clinical algorithm. This means that the predictive performance in another cultural environment may deviate from the one reported in this study. Therefore, results in other countries and environments of repeating our approach is required. Another limitation regards the evaluation: applying bootstrapping to the whole training set and repeating the whole learning procedure with the validation set (instead of only applying bootstrapping on the predicted probabilities on the test set) would have accounted also for the variability in choices of the training, validation, and test sets. This, however, would be computationally prohibitively expensive in our case and not scale. We excluded patients with very long notes that did not fit in the GPU memory. While this was necessary to run the model, this preprocessing step may concern the most ‘difficult’ cases. These are patients that may suffer from many diseases or have complex psychosocial issues. However, the number of patients excluded is minimal (0.03%). An alternative would be to discard more notes from each patient in order to fit the complex cases into memory. However, such solution would have resulted in a higher information loss overall (more text excluded), that is why we opted to exclude patients with most text. One of the limitations of neural networks for text analysis is that the function linking predictors to outcome is essentially a ‘black box’; we do not know which texts, words or phrases are most associated with the outcome. If implemented in daily practice, it might be challenging to convince GPs and patients of the validity of the process. Related to this limitation, a general word of caution for evaluating clinical prediction models is that prediction performance is not the only factor to consider when evaluating models for healthcare applications [38]. Finally, further external validation of the model is needed before its adoption in clinical practice. Using data from other centers would also enable to test the model on a larger number of cancer patients. In this study, the testing cohort is 20% of the patients, which means that it includes only about 143 out of 712 lung cancer patients.

### 4.4. Implications

For researchers and practitioners working with prediction models and decision support systems, we showed the promise of leveraging the order, hierarchy, and context among words and sentences in real-world primary-care clinical notes together with a powerful optimization strategy based on target replication. The model’s probability cut-offs can be fine-tuned to fit several settings, each with its own implications for clinicians. Positive predictive values, sensitivity and specificity of our algorithm can be adapted to the specific clinical context. For primary care practice, we developed a model able to indicate a relevant risk of lung cancer several months earlier. For primary care clinicians, the implication is helping them achieve one of their main objectives: earlier recognition of a substantial risk of serious disease. For society it might help to improve the prognosis of a mostly lethal disease, without increase in costs.

### 4.5. Future Research

Before applying the models to clinical practice, the models should be externally validated to assess the generalizability of the performance. This will also allow testing the models on a larger number of lung cancer patients. More data from multiple centers may increase model performance, give insight into external validation and facilitate adaptation to the local context. Besides the methodological, technical and epidemiological challenges, also ethical, societal, legal and privacy issues should be considered in future research. Important questions on how the model should be implemented in a decision support system, including when it is triggered and how it provides advice, need to be investigated. Finally, this approach could be applied and evaluated in other types of cancer and diseases.

## 5. Conclusions

We demonstrated that capturing the inherent order, hierarchy, and context of words and sentences within clinical notes yielded excellent predictive performance. The optimization strategy based on target replication was key in developing a performant model. To detect one patient with lung cancer 5 months before the current diagnosis (4 months before referral), 29 high-risk patients would need additional diagnostic testing. Incorporating clinical variables in addition to text does improve the results. Such an AI-based model, leveraging both clinical variables and textual data, has the potential to improve early detection of lung cancer. However, further work is needed to externally validate the model and assess its suitability in clinical practice before clinical implementation.

## Figures and Tables

**Table 1:** Example of a generic patient note of 2 sentences. The words “S” “O” “E” and “P” are part of the text.

Sentence 1	*S word1, word2, word3 … O word1, word2, word3…* *E* *… P …*
Sentence 2	*S word1 word2, … O word1, … E word1, … P word1, ….*

**Table 2:** Descriptive statistics of our study population, stratified by lung cancer diagnosis. IQR stands for interquartile range.

	Non-Lung Cancer	Lung Cancer	Total
N (%)	182,300 (99.61)	712 (0.39)	183,012 (100.00)
Age—Median (IQR)	52 (40–64)	68 (61–76)	52 (40–64)
Number of consultations per patient—Mean (SD)	140 (170)	160 (170)	140 (170)
Number of unique ICPC codes per patient—Mean (SD)	12 (32)	11 (28)	12 (32)

**Table 3:** Performance of HAN-Text and HAN-Combined models on the test set. AUROC, AUPRC, and Brier score with 95% confidence intervals are reported.

Model	AUROC	AUPRC	Brier Score (×100)
HAN-Text	0.908 (0.886, 0.932)	0.0773 (0.030, 0.103)	0.37 (0.31, 0.43)
HAN-Combined	0.913 (0.892, 0.935)	0.048 (0.028, 0.062)	0.38 (0.32, 0.44)

**Table 4:** Performance of the HAN-Text and HAN-Combined models on the test set. Sensitivity, specificity, PPV, and NPV with 95% confidence intervals are reported.

Model	Sensitivity	Specificity	PPV	NPV
HAN-Text	0.852 (0.839, 0.866)	0.779 (0.694, 0.855)	0.0200 (0.015, 0.024)	0.999 (0.999, 0.999)
HAN-Combined	0.928 (0.919, 0.939)	0.657 (0.585, 0.740)	0.034 (0.024, 0.043)	0.999 (0.998, 0.999)

**Table 5:** Summary of the main characteristics of the compared models.

Model	Hierarchical	Attention	Target Replication
HAN-Text	●	●	●
HN	●		●
LSTM			●
PSGNN			
CNN	●		●

**Table 6:** Performance of the additional text-based models. AUROC, AUPRC, and Brier score with 95% confidence intervals are reported. We also report the performance of the HAN-Text model for comparison.

Model	AUROC	AUPRC	Brier Score (×100)
HAN-Text	0.908 (0.886, 0.932)	0.0773 (0.030, 0.103)	0.37 (0.31, 0.43)
HN	0.876 (0.847, 0.910)	0.060 (0.027, 0.083)	0.37 (0.32, 0.43)
LSTM	0.872 (0.841, 0.905)	0.042 (0.015, 0.054)	0.39 (0.32, 0.44)
PSGNN	0.870 (0.776, 0.847)	0.017 (−0.003, 0.023)	2.10 (1.97, 2.22)
CNN	0.813 (0.782, 0.844)	0.029 (−0.001, 0.043)	0.38 (0.31, 0.44)

**Table 7:** Performance of the additional text-based models. Sensitivity, specificity, PPV and NPV with 95% confidence intervals are reported. We also report the performance of the HAN-Text model for comparison.

Model	Sensitivity	Specificity	PPV	NPV
HAN-Text	0.852 (0.839, 0.866)	0.779 (0.694, 0.855)	0.0200 (0.015, 0.024)	0.999 (0.999, 0.999)
HN	0.963 (0.958, 0.969)	0.500 (0.403, 0.586)	0.049 (0.034, 0.061)	0.998 (0.997, 0.999)
LSTM	0.830 (0.809, 0.856)	0.786 (0.714, 0.861)	0.018 (0.013, 0.022)	0.999 (0.998, 0.999)
PSGNN	0.898 (0.889, 0.907)	0.500 (0.415, 0.583)	0.019 (0.013, 0.023)	0.998 (0.997, 0.999)
CNN	0.816 (0.798, 0.836)	0.614 (0.526, 0.708)	0.013 (0.010, 0.016)	0.998 (0.998, 0.999)

## Data Availability

The data underlying this article were provided by the Academic General Practitioner’s Network of Amsterdam UMC. For privacy reasons, the data cannot be made publicly available. Data are available from the research network for researchers who meet the criteria for access to confidential data. Our code is available at https://bitbucket.org/aumc-kik/aidoc, accessed on 26 March 2025.

## Supplementary Materials

The following supporting information can be downloaded at: https://www.mdpi.com/article/10.3390/cancers17071151/s1.

## Appendix A

Models

In addition to the two HAN-based models, we developed four other models. For these additional models, only the GP free-text consultation notes were used.

The first model is a hierarchical network (HN). It is a variation of the HAN-Text model, where the attention layers have been removed.

The second model is a flat LSTM model, which does not exploit the hierarchy of words and sentences. Here, a BiLSTM layer represents a patient note (visit) and the final patient representation averages the representation of each note of the same patient.

For the third model (PSGNN), first a representation of each word and phrase was obtained using a Phrase Skip-Gram (PSG) algorithm [4]. A phrase is a list of adjacent words that frequently appear such as “heart failure”. Phrases of 3-g (three words at a time) were used because they proved better in our previous study [4]. The embeddings were then fed to a neural network prediction model through a hidden layer with a Rectified Linear Unit (ReLU) activation function to allow for interactions between the embeddings. The output of this layer was then averaged to produce a single embedding that represents all the patient text. The elements of this embedding constitute the text predictors. Finally, a logistic regression model was used to predict the probability of lung cancer. The architecture of the PGSNN model is shown in Figure A1.

For the fourth model, we developed a convolutional neural network (CNN). As performed by Grnarova et al. for mortality prediction [9], we adopted a two-layer architecture. The first layer independently maps sentences (visits) to sentence embeddings. The second layer combines sentences into a single patient representation. For both levels, we use convolutional neural networks (CNNs) with max-pooling. We also use target replication, which replicates the loss at intermediate steps (i.e., for intermediate visits), computes an individual lung cancer probability for every visit, and incorporates additional loss terms for each into our final loss. For the CNN model, we use unigrams (one word at a time).

Confusion Matrices of the HAN Models

Table A1 and Table A2 show the confusion matrices of the HAN-Text and HAN-Combined models.

**Table A1:** Confusion matrix of the HAN-Text model. The probability cut-off is 0.004 (equal to the prevalence of lung cancer).

		Actual	
		Non Lung Cancer	Lung Cancer
Predicted	Non lung cancer	30,992	31
	Lung cancer	5402	109

**Table A2:** Confusion matrix of the HAN-Combined model. The probability cut-off is 0.004 (equal to the prevalence of lung cancer).

		Actual	
		Non Lung Cancer	Lung Cancer
Predicted	Non lung cancer	33,766	48
	Lung cancer	2628	92

Model Hyperparameters

The following hyperparameters were used for Word2Vec: use skip-gram, an embedding dimension of 300, a window size of 5, using negative sampling (k = 5), a threshold of 0.0001 frequency for word subsampling and a (lower) minimum word count of 3, to keep more rare misspelling variants. Word subsampling is performed to deal with the imbalance between rare and frequent words. Frequent words (e.g., ‘the’, ‘a’, et cetera) usually provide less information than rare words. Specifically, we performed word down sampling (i.e., more frequent words are down sampled) where words are discarded with a probability inverse to their frequency: this strategy aggressively subsamples words whose frequency is greater than a threshold t (here: 0.0001) while preserving the ranking of the frequencies [13]. For all models, we minimized binary cross entropy loss with the Adam optimizer with gradient accumulation and a learning rate of 0.001 and used a dropout of 0.2. For the PSGNN model, we used early stopping on the validation set with a maximum number of 30 and 15 epochs, respectively, a batch size of 16 and 4 gradient steps [39]. For the HAN-Text and HAN-Combined models, we used early stopping on the validation set with a maximum number of 15 epochs, a batch size of 1 and 64 gradient accumulation steps [39]. The size of the embeddings of the structured variables was 32. Target replication was set with a replication factor, *λ*, of 0.001 (see Figure 1 in the main article). For the CNN model we used convolutional neural networks (CNNs) with max-pooling, word kernels of sizes of 3, 4, and 5, sentence kernels of size 3, and a batch size of 1 and 64 gradient accumulation steps.

Running Time and Cost

The running time of training each HAN model (HAN-Text and HAN-Combined, respectively) was about 16 h. Models were trained with a Standard NC6 v3 (https://learn.microsoft.com/en-us/azure/virtual-machines/sizes/gpu-accelerated/nc-series?tabs=sizebasic, last accessed on 26 March 2025) virtual machine in the trusted research computational environment myDRE (https://andrea-cloud.com/, last accessed on 26 March 2025), which is based on Microsoft Azure (https://azure.microsoft.com, last accessed on 26 March 2025). A standard NC6 virtual machine is equipped with 6 virtual CPU Intel Xeon E5-2690 v3, 112 GB RAM and a GPU NVIDIA Tesla K80 GPU with 24 GB of dedicated memory. The total cost of running these models including preprocessing and evaluation was about 70€ per model. The other LSTM-based models had similar running time and costs. Training CNN models was slightly faster and therefore cheaper.
